# Is Body Dissatisfaction a Risk Factor for Diabulimia and How Is It Assessed? A Rapid Systematic Review

**DOI:** 10.1192/bjo.2024.217

**Published:** 2024-08-01

**Authors:** Navaneeth Natarajan, Shaymaa Obousy

**Affiliations:** King's College London, London, United Kingdom

## Abstract

**Aims:**

Diabulimia is an increasingly used term referring to “an eating disorder (ED) with type 1 diabetes”. It is difficult to detect and presents in multiple ways, which can potentially include feelings of body dissatisfaction (BD), which in itself is a complex symptom to quantify clinically.

This rapid systematic review aimed to identify whether feelings of BD are a risk factor for diabulimia by researching if and how BD is assessed in patients with the condition.

**Methods:**

A rapid systematic review was undertaken. A literature review was performed on Ovid Medline (all) and Ovid Embase databases using search terms for Type 1 Diabetes, ED, and BD and looked at cross-sectional studies only. One reviewer performed the literature search and screened titles and abstracts. Out of 589 papers screened, four papers met the inclusion criteria. These papers then went through critical appraising using the Appraisal tool for Cross-Sectional Studies, with all papers showing mid-level quality clearing 16 to 17/20 questions. Therefore, data was extracted from all of them.

**Results:**

All four papers came from different countries and used a wide range of sample sizes (43–477).

There was widespread heterogeneity between the data collected in each study due to the various tools used to identify BD, paired with differences in analysing extracted data.

To ensure transparency and quality of the results provided, the Synthesis Without Meta-analysis tool was used. Three studies looked at effects on adolescents and three had a higher proportion of females. All papers used previously established and tested BD screening methods. Two papers found female diabetics were more likely to have BD symptoms, and one paper saw that males were more at risk. All four papers concluded that BD had some correlations with one or more aspects of diabetes and/or other ED symptoms related to diabulimia. Two commented on positive correlations between BD and HbA1c levels and one commented on BD symptoms and insulin restriction trending together. Two papers also saw BD symptoms and depressive symptoms correlating in patients as well.

**Conclusion:**

All four studies showed that BD was related to diabulimia, both from a psychological and diabetic perspective, and most highlighted how BD manifested between the different sexes of diabetics. This review highlights the need for more standardised and comprehensive BD questionnaires to draw out key signs of EDs in diabetics that could improve screening, detection and management of diabulimia.

